# Clinical relevance of circulating MACC1 and S100A4 transcripts for ovarian cancer

**DOI:** 10.1002/1878-0261.12484

**Published:** 2019-04-15

**Authors:** Theresa Link, Jan Dominik Kuhlmann, Dennis Kobelt, Pia Herrmann, Yana D. Vassileva, Michael Kramer, Kerstin Frank, Maren Göckenjan, Pauline Wimberger, Ulrike Stein

**Affiliations:** ^1^ Department of Gynecology and Obstetrics Medical Faculty University Hospital Carl Gustav Carus Technische Universität Dresden Germany; ^2^ National Center for Tumor Diseases (NCT), Dresden, Germany; German Cancer Research Center (DKFZ), Heidelberg, Germany; Faculty of Medicine and University Hospital Carl Gustav Carus, Technische Universität Dresden, Dresden, Germany; Helmholtz‐Zentrum Dresden – Rossendorf (HZDR) Dresden Germany; ^3^ German Cancer Consortium (DKTK) Dresden and German Cancer Research Center (DKFZ) Heidelberg Germany; ^4^ Experimental and Clinical Research Center Charité – Universitätsmedizin Berlinand Max‐Delbrück‐Center for Molecular Medicine in the Helmholtz Association Berlin Germany; ^5^ German Cancer Consortium (DKTK) Berlin and German Cancer Research Center (DKFZ) Heidelberg Germany; ^6^ Medizinische Klinik und Poliklinik I Medical Faculty and University Hospital Technische Universität Dresden Germany; ^7^ DRK‐Blood Donor Service ITM Plauen Germany

**Keywords:** blood‐based biomarker, MACC1, ovarian cancer, prognosis, S100A4, survival

## Abstract

Metastasis‐associated in colon cancer 1 (MACC1) and S100 calcium‐binding protein A4 (S100A4) are prominent inducers of tumor progression and metastasis. For the first time, we systematically tracked circulating serum levels of MACC1 and S100A4 transcripts in the course of surgery and chemotherapy and analyzed their clinical relevance for ovarian cancer. MACC1 and S100A4 transcripts were quantified in a total of 318 serum samples from 79 ovarian cancer patients by RT‐qPCR and digital droplet PCR, respectively. MACC1 and S100A4 transcripts were significantly elevated in serum of ovarian cancer patients, compared to healthy controls (*P* = 0.024; *P* < 0.001). At primary diagnosis, high levels of MACC1 or S100A4 correlated with advanced FIGO stage (*P* = 0.042; *P* = 0.008), predicted suboptimal debulking surgery and indicated shorter progression‐free survival (PFS;* P* = 0.003; *P* = 0.001) and overall survival (OS;* P* = 0.001; *P* = 0.002). This is the first study in ovarian cancer to propose circulating MACC1 and S100A4 transcripts as potential liquid biopsy markers.

**Abbreviations**
ASA: American Society of Anesthesiologists
AUC: area under the curve
BRCA1: breast cancer 1, early onset
CA125: cancer‐antigen 125
CI: confidence interval
CRC: colorectal cancer
ddPCR: droplet digital polymerase chain reaction
ED: estimated difference
EMT: epithelial‐to‐mesenchymal transition
FIGO: *Fédération Internationale de Gynécologie et d'Obstétrique*
HGF: hepatocyte growth factor
HR: hazard ratio
MACC1: Metastasis‐associated in colon cancer 1
MET: MET proto‐oncogene, receptor tyrosine kinase
OS: overall survival
PARP: poly(ADP‐ribose) polymerase
PFS: progression‐free survival
ROC: receiver operating characteristic
RT‐qPCR: real‐time quantitative polymerase chain reaction
S1004A: S100 calcium‐binding protein A4

## 1 Introduction

Ovarian cancer is the leading cause of death among female malignancies. At primary diagnosis, approximately 70% of all ovarian cancer patients already present with advanced tumor stages (Goodman *et al*., 2003). Standard treatment of advanced ovarian cancer is primary radical surgery aiming at macroscopic complete tumor resection followed by platinum‐ and paclitaxel‐based chemotherapy, which prolongs progression‐free survival (PFS) and overall survival (OS) (du Bois *et al*., 2005; Karam *et al*., 2017). Postoperative residual tumor load is one of the most important prognostic factors in advanced ovarian cancer (Wimberger *et al*., 2010). Despite improved primary radical surgery and the implementation of innovative targeted therapies into standard treatment of ovarian cancer, such as anti‐angiogenetic therapy with bevacizumab or PARP inhibitors, ovarian cancer patients still have a poor overall prognosis (Burger *et al*., 2011; Ledermann *et al*., 2014). Considering this clinical challenge, it would be highly desirable to establish additional blood‐based biomarkers, which can predict recurrence risk and prognosis.

In previous studies, we focused on two human metastasis‐inducing genes: metastasis‐associated in colon cancer 1 (MACC1) and S100 calcium‐binding protein A4 (S100A4). MACC1 was newly identified and named by our group (Stein *et al*., 2009a). MACC1 is a key regulator of HGF‐MET signaling and a decisive driver of metastasis in human colorectal cancer (CRC) (Arlt and Stein, 2009; Stein *et al*., 2009a,2009b). MACC1 promotes metastasis as a regulator of further metastasis‐associated genes by acting as a transcription factor and/or via protein–protein interactions. MACC1 induces fundamental processes like proliferation, migration, and invasiveness resulting in tumor progression and metastasis in xenografted and transgenic mouse models (Lemos *et al*., 2016; Mudduluru *et al*., 2017; Stein *et al*., 2010). We and others reported that MACC1 levels in early primary CRC predict metastasis formation and poor survival (Ilm *et al*., 2015; Koelzer *et al*., 2015; Nitsche *et al*., 2012; Stein *et al*., 2009a). These findings have been confirmed by more than 190 successive publications for more than 20 other human solid malignancies.

S100A4 belongs to the family of S100 proteins, which are a highly similar group of small Ca^2+^‐binding proteins with a molecular mass of 10–12 kDa (Dahlmann *et al*., 2016; Zimmer *et al*., 2013). S100 proteins are involved in numerous different cellular functions, such as proliferation, differentiation, or apoptosis (Donato *et al*., 2013). Enhanced cell growth and motility, associated with elevated S100A4 expression, was shown to increase the metastatic potential of cancer cells among a variety of malignancies, such as breast cancer, lung cancer, or CRC (Bresnick *et al*., 2015). S100A4 was identified as transcriptional target of the Wnt/β‐catenin signaling pathway. Targeting this pathway decreased S100A4 expression and reduced metastasis formation in a CRC mouse model (Sack *et al*., 2011; Stein *et al*., 2006).

Transferring this to a liquid biopsy approach, we have recently established a sensitive blood‐based assay for detecting the transcripts of MACC1 and S100A4 in serum/plasma. We showed that (a) MACC1 mRNA transcripts are detectable in plasma of patients with CRC or gastric cancer, (b) they are significantly elevated compared to healthy controls, and that (c) high levels of circulating MACC1 transcripts indicate poor survival (Ashktorab *et al*., 2016; Burock *et al*., 2015; Lederer *et al*., 2015; Rohr *et al*., 2017; Stein *et al*., 2012). Furthermore, we have confirmed the diagnostic and prognostic value of S100A4 transcripts in plasma of patients with CRC or gastric cancer, particularly by combined assessment, together with MACC1 transcripts (Burock *et al*., 2015; Stein *et al*., 2011, 2012).

Oncogenic function of MACC1 and S100A4 has likewise been proposed for ovarian cancer. It was reported that both genes are overexpressed in ovarian cancer and this overexpression was associated with increased migration, chemoresistance, and epithelial‐to‐mesenchymal transition (EMT) (Kikuchi *et al*., 2006; Li *et al*., 2015; Yan *et al*., 2016; Yu *et al*., 2017; Zhang *et al*., 2016). However, clinical relevance of MACC1 and S100A4 transcripts as potential blood‐based biomarkers for ovarian cancer patients is completely unknown. Therefore, we inquired (a) whether we can detect MACC1 and S100A4 mRNA transcripts in serum of ovarian cancer patients at a level that significantly differs from healthy controls and (b) whether MACC1 and S100A4 transcript levels at primary diagnosis and in the course of surgery and adjuvant chemotherapy could have the potential as liquid biopsy markers for ovarian cancer.

## 2 Materials and methods

### 2.1 Patient characteristics

The study methodologies conformed to the standards set by the Declaration of Helsinki. Our study was conducted at the Department of Gynecology and Obstetrics at the Carl Gustav Carus University of Dresden, TU Dresden, Germany. In this study, a total of 79 patients with histologically confirmed primary epithelial ovarian cancer were enrolled. Informed written consent was obtained from all patients, and the study was approved by the Local Research Ethics Committee in Dresden (EK74032013). The patients’ clinical data are summarized in Table 1. Tumors were classified according to the WHO classification of tumors of the female genital tract. Grading was conducted using the grading system proposed by Silverberg (Silverberg, 2000), and tumor staging was classified according to the *Fédération Internationale de Gynécologie et d'Obstétrique* (FIGO) (FIGO Committee on Gynecologic Oncology 2009). The whole study population received primary radical surgery aiming at macroscopic complete tumor resection followed by platinum‐ and paclitaxel‐based adjuvant chemotherapy. Bevacizumab, which is approved for patients with a tumor stage of at least FIGO IIIb, was additionally administered to 66% of all patients.

**Table 1:** Patient characteristics

Total number of patients	79
Age	Median 60 years, (21–82 years)
FIGO stage	
I‐II	16 (20%)
III‐IV	63 (80%)
Grading	
I	3 (4%)
II–III	76 (96%)
Residual tumor	
Macroscopic complete resection	36 (46%)
Any residual tumor	43 (54%)
Histologic type	
Serous	63 (80%)
Others	16 (20%)
Recurrence	
PFS	Median 556 days (33–1637 days)
No relapse	38 (48%)
Relapse	41 (52%)
Survival	
OS	Median 655 days (33–1637 days)
Alive	57 (72%)
Dead	22 (28%)

OS, overall survival; PFS, progression‐free survival.

### 2.2 Serum preparation and RNA isolation

Following blood withdrawal with a 7.5‐mL S‐Monovette^®^ (Sarstedt AG & Co., Nuembrecht, Germany), obtained blood samples were incubated at room temperature for at least 30 min to allow complete blood coagulation. Within 1 h post‐blood drawing, serum was prepared by centrifugation for 8 min at 1800 ***g*** at room temperature. The obtained cell‐free serum fraction was immediately frozen at −80 °C until further processing. Unnecessary freeze–thaw cycles were avoided. Samples were blinded so that neither time of blood drawing nor any other information was disclosed during analysis. Samples were thawed on ice and immediately processed after complete thawing. For isolation of total RNA, the High Pure Viral RNA Kit (Roche Diagnostics, Mannheim, Germany) was used according to the manufacturer's instruction as described previously (Ashktorab *et al*., 2016; Burock *et al*., 2015; Stein *et al*., 2011, 2012). Briefly, 350 μL serum was mixed with binding buffer, supplemented with Poly(A) carrier RNA (50 μg·mL^−1^). After RNA binding to the columns and washing steps, RNA was eluted in nuclease‐free water.

### 2.3 Real‐time quantitative polymerase chain reaction

For reverse transcription 1xPCR buffer II (Life Technologies, Carlsbad, CA, USA), 5 mm MgCl_2_ (Life Technologies), 1 mm of each dNTP (Roboklon, Berlin, Germany), 2.5 μm random hexamers (Life Technologies), 1 U·μL^−1^ RNase inhibitor (Applied Biosystems, Foster City, CA, USA), and 2.5 U·μL^−1^ MuLV reverse transcriptase (Applied Biosystems) were used to transcribe isolated serum RNA. Reaction was performed in a PCR cycler at 23 °C for 15 min, 42 °C for 45 min, and 95 °C for 5 min in a volume of 20 μL. MACC1 transcript levels were quantified using a Roche LightCycler 480 (Roche) and GoTaq^®^ Probe qPCR Master Mix (Promega Corporation, Madison, WI, USA) following manufacturer's instructions. Briefly, 1x master mix supplemented with 0.5 μm of each primer (fw: TTC TTT TgA TTC CTC Cgg TgA, rev: ACT CTg ATg ggC ATg TgC Tg) and 0.3 μm probes (gCA gAC TTC CTC AAg AAA TTC Tgg AAg ATC TA –FL, LC640‐AgT gTT TCA gAA CTT CTg gAC ATT TTA gAC gA‐P; syntheses of primers and probes: BioTeZ and TIB MolBiol, Berlin, Germany) and water were mixed in duplicate with 2 μL cDNA to a final volume of 10 μL. PCR was performed at 95 °C for 2 min followed by 40x 95 °C for 3 s, 60 °C for 5 s, and 60 °C for 25 s with data acquisition after the primer annealing step at the first 60 °C incubation step and a melting curve from 40 °C to 95 °C. MACC1 mRNA expressions are given as percentage of the mRNA expression of a calibrator sample, which was set 100%. Quantification was performed using serial dilutions of a calibrator sample. The calibrator cDNA derived from the cell line SW620 [authentication of the cell line by short tandem repeat (STR) genotyping, German Collection of Microorganisms and Cell Cultures, Braunschweig, Germany]. Each sample was run and calculated in duplicate, and mean values are indicated. Data analysis was performed using the lightcycler software.

### 2.4 Droplet digital polymerase chain reaction

S100A4 transcript levels were quantified using a Bio‐Rad QX200 Droplet Digital PCR System (Bio‐Rad Laboratories GmbH, Munich, Germany) and droplet digital polymerase chain reaction (ddPCR) Supermix for Probes (Bio‐Rad) following manufacturer's instructions. Briefly, 1x master mix supplemented with primer probe mix (qHsaCEP0055305, Bio‐Rad) and cDNA. Droplet generation for ddPCR was done following manufacturer's instructions using the QX200 droplet generator. Briefly, 20 μL PCR mix and 70 μL droplet generator oil are given in the respective cavities of the droplet generator cartridges (Bio‐Rad). After droplet generation, 40 μL droplet suspension was pipetted into twin.tec 96‐well PCR plates (Eppendorf, Hamburg, Germany). PCR was performed at 95 °C for 10 min followed by 39x 95 °C for 30 s and 60 °C for 60 s and 98 °C for 10 min using a T100 thermal cycler. Droplet quantification was performed in the QX200 droplet reader. The system counts all generated droplets and detects the amount of PCR product‐positive (fluorescent) droplets. Poisson correction of generated droplet amount and data analysis was done using the quanta life (Bio‐Rad) software.

### 2.5 Statistical analysis

Statistical analysis was performed using python Version 3.6.4 (Python Software Foundation, Delaware, USA) and graphpad prism version 7 for Windows (GraphPad Software, La Jolla, CA, USA) and R‐3.4.4 (Free Software Foundation's GNU General Public License). For this data analysis, *P*‐values < 0.05 were considered to be statistically significant. Evaluation of circulating MACC1 and S100A4 was performed using a nonparametric two‐sided Mann–Whitney‐*U*‐Test. To analyze, if MACC1 and S100A4 can discriminate between ovarian cancer patients and healthy controls, receiver operating characteristic (ROC) curve analysis was performed. For estimating the correlation between MACC1, S100A4, and CA125, Spearman's rank correlation test was performed. The change of MACC1 and S100A4 transcripts levels during treatment was analyzed with the two‐sided Wilcoxon rank‐sum test for paired data. Associations of MACC1 and S100A4 with clinicopathological parameters were analyzed by the nonparametric two‐sided Mann–Whitney‐*U*‐Test. To evaluate prognostic relevance of MACC1 and S100A4 transcript levels, univariate Cox regression was used (with or without time dependency). A multivariate Cox regression including established risk factors for ovarian cancer (age, FIGO stage, residual tumor, histology, state of health and amount of ascites) was additionally performed. All models were tested for both PFS und OS. In addition, Kaplan–Meier analysis and the log‐rank test were performed.

## 3 Results

### 3.1 Serum levels of MACC1 and S100A4 transcripts in ovarian cancer patients and their dynamics in the course of surgery and adjuvant chemotherapy

We analyzed the levels of serum MACC1 and S100A4 transcripts in a cohort of clinically documented ovarian cancer patients at primary diagnosis (*n* = 77) and compared it to the levels in serum of healthy controls (*n* = 25). Moreover, we tracked the levels of both transcripts in the course of primary surgery and adjuvant chemotherapy, represented by four additional longitudinal follow‐up samples, obtained (a) one week after primary surgery (*n* = 72), (b) before platinum‐based chemotherapy (*n* = 63), (c) after the third cycle of chemotherapy (*n* = 53), and (d) after completion of chemotherapy (*n* = 53).

**Figure 1:**
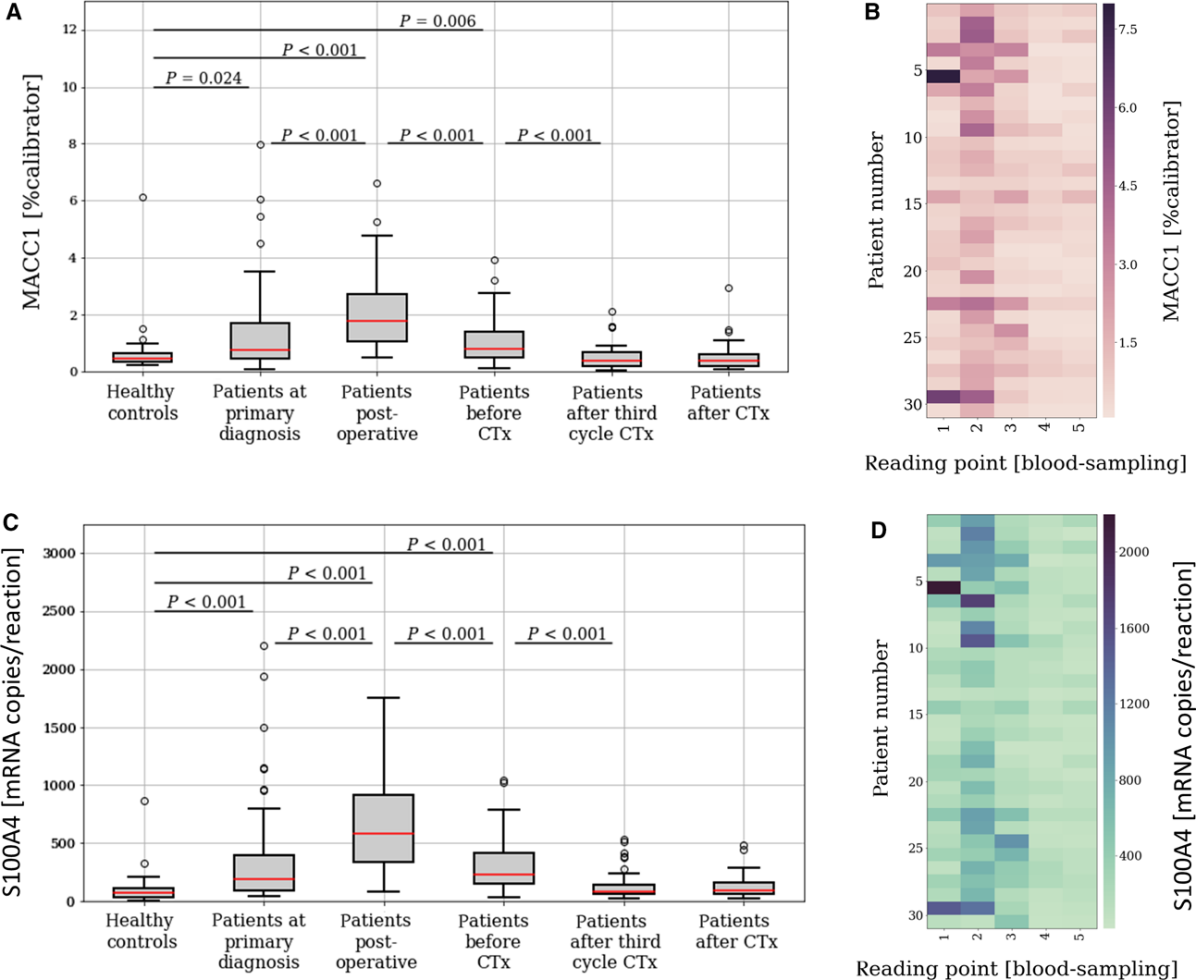
The dynamics of circulating MACC1 and S100A4 transcripts in the course of surgery and adjuvant chemotherapy. Level of circulating MACC1 (A + B) and S100A4 (C + D) serum transcripts in ovarian cancer patients at (1) primary diagnosis (*n* = 77) and among four longitudinal serum samples per patient, obtained (2) one week after primary surgery (*n* = 72), (3) before platinum‐based chemotherapy (*n* = 63), (4) after third cycle of chemotherapy (*n* = 53), and (5) after the completion of chemotherapy (*n* = 53), compared to healthy controls (*n* = 25). The whiskers are drawn down to the 10th percentile and up to the 90th percentile. Values below or above the whiskers are shown as individual dots. The median is plotted at the line in the middle of the box. For those patients with a complete set of all 5 blood samples (*n* = 31), heat maps were drawn, visualizing the individual course of MACC1 (B) or S100A4 (D). *P*‐values, according to the two‐sided Wilcoxon test for paired samples and according to the two‐sided Mann–Whitney‐*U*‐Test for independent samples, are indicated.

Compared to healthy controls, the level of MACC1 transcripts was significantly upregulated at primary diagnosis of ovarian cancer [estimated difference (ED) = 0.22, CI = (0.03–0.49); *P* = 0.024; Fig. 1A + B]. ROC curve analysis revealed that the level of MACC1 at primary diagnosis discriminates ovarian cancer patients from healthy controls with an area under the curve (AUC) of 0.65 [CI = (0.53–0.77); Fig. S1]. The level of S100A4 was likewise upregulated at primary diagnosis [ED = 108, CI = (59–188); *P* < 0.001) and discriminated cancer patients from healthy controls with an AUC of 0.81 [CI = (0.70–0.91); Fig. 1C + D, Fig. S1]. During surgery and adjuvant chemotherapy, progression of MACC1 and S100A4 transcript levels was highly comparable. One week after surgery, both transcripts strongly increased [MACC1: ED = 0.75, CI = (0.45–1.05); *P* < 0.001; S100A4: ED = 298, CI = (204–411); *P* < 0.001] and then declined in the course of adjuvant chemotherapy, gradually approaching the level of healthy controls over time. Until beginning of chemotherapy, both transcripts were still significantly elevated compared to healthy controls (Fig. 1A + C).

**Figure 2:**
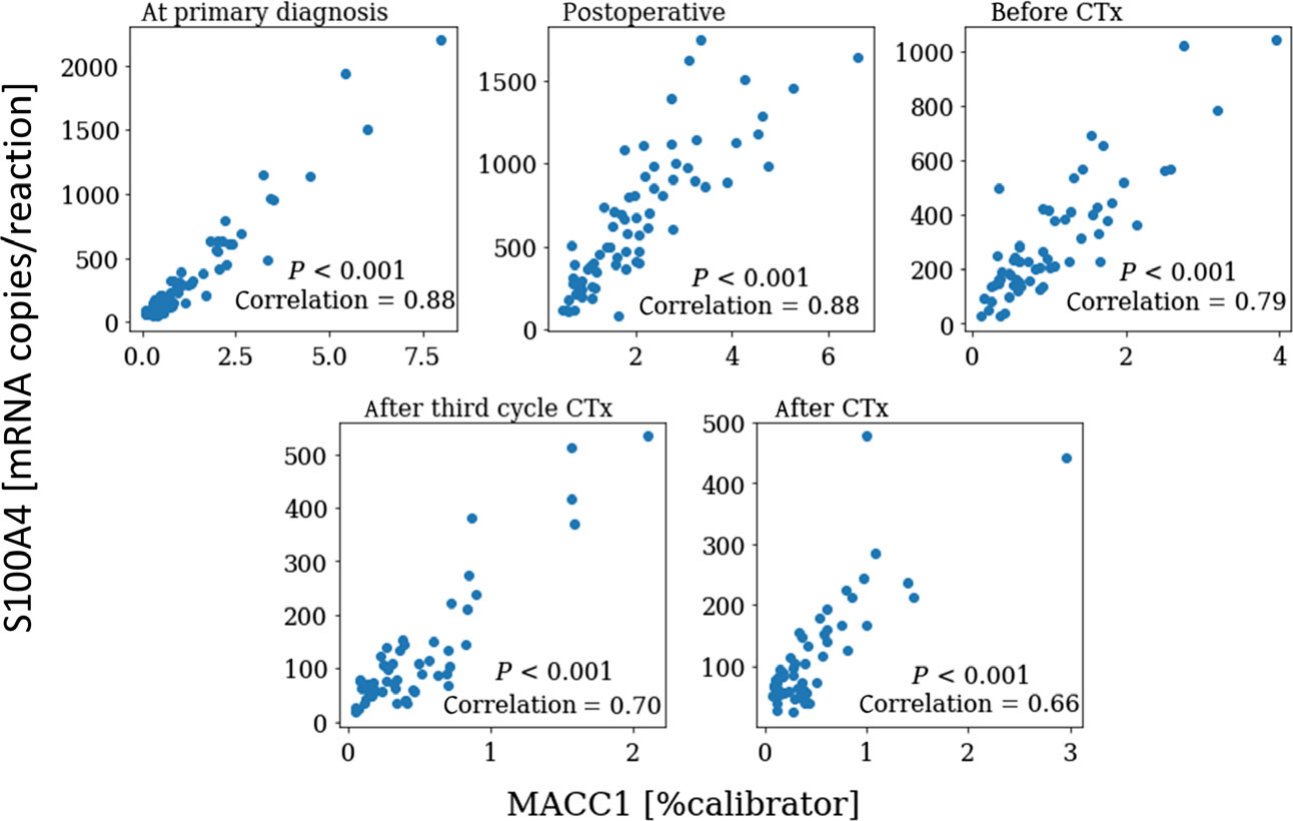
Correlation between circulating MACC1 and S100A4 transcript levels. Coherence of MACC1 and S100A4 values for all blood samples taken at primary diagnosis and at four defined reading points in the course of primary surgery and adjuvant chemotherapy (1: at primary diagnosis; 2: one week after primary surgery; 3: before platinum‐based chemotherapy; 4: after third cycle of chemotherapy; 5: after the completion of chemotherapy). Correlation coefficients and *P*‐values, according to Spearman's rank‐order test, are indicated.

There was a strong correlation between circulating MACC1 and S100A4 transcript levels at primary diagnosis and across all longitudinal follow‐up samples [correlation coefficients (*r*): 0.877, 0.876, 0,786, 0.703, 0.661, respectively; *P* < 0.001; Fig. 2]. Interestingly, there was no correlation between the level of MACC1 and the clinically established serum marker CA125 at any of the investigated follow‐up samples. The same trend was true for S100A4; there was merely a weak correlation between S100A4 and CA125 before chemotherapy (*r* = 0.269; *P* = 0.047) and after completion of chemotherapy (*r* = 0.311; *P* = 0.029), but especially at primary diagnosis and during chemotherapy, CA125 and S100A4 serum levels were independent from each other.

Conclusively, circulating MACC1 and S100A4 transcripts are significantly elevated in serum of ovarian cancer patients and show a high correlation in the course of surgery and chemotherapy, which is mostly independent from CA125 serum levels. Compared to MACC1, S100A4 levels allow superior discrimination between ovarian cancer patients and healthy controls.

### 3.2 Association of circulating MACC1 and S100A4 transcript levels with the patient's clinicopathological parameters

**Figure 3:**
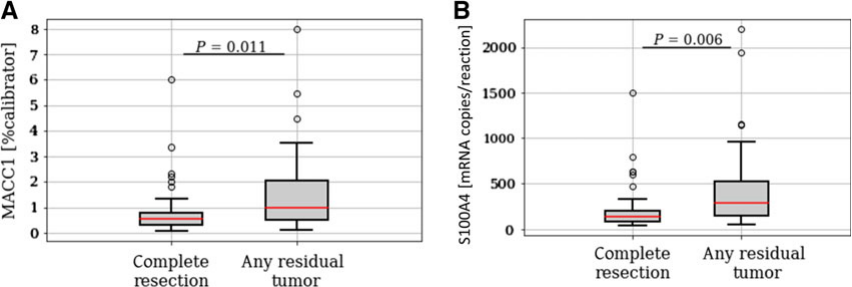
Association of circulating MACC1 and S100A4 transcript levels with debulking efficiency. Circulating MACC1 (A) and S100A4 (B) transcript levels at primary diagnosis in ovarian cancer patients with a macroscopic complete tumor resection vs. patients with any residual tumor. The whiskers are drawn down to the 10th percentile and up to the 90th percentile. Values below or above the whiskers are shown as individual dots. The median is plotted at the line in the middle of the box. *P*‐values, according to two‐sided Mann–Whitney‐*U*‐Test, are indicated.

We related serum levels of circulating MACC1 and S100A4 transcripts with the patient's clinicopathological data. Higher levels of MACC1 and S100A4 transcripts at primary diagnosis correlated with advanced disease, indicated by a higher FIGO stage (*P *= 0.046, *P *= 0.008, respectively) and an increased load of malignant ascites [MACC1: ED = 0.37, CI = (0.11–0.73); *P* = 0.008; S100A4: ED = 118, CI = (43–224); *P* = 0.002). Interestingly, higher levels of both candidate transcripts at primary diagnosis were associated with residual tumor burden left after primary debulking [MACC1: ED = 0.34, CI = (0.07–0.70); *P* = 0.011; S100A4: ED = 115, CI = (24–203); *P* = 0.006; Fig. 3).

Collectively, high circulating MACC1 and S100A4 levels in serum of ovarian cancer patients correlate with advanced disease and predict suboptimal primary debulking surgery without achieving macroscopically complete tumor resection.

### 3.3 Prognostic relevance of circulating MACC1 and S100A4 transcripts

In order to study the prognostic relevance of serum MACC1 and S100A4 transcripts at primary diagnosis, we performed univariate Cox regression analysis. A MACC1 level increase of 1 relative unit was associated with a 40% increase in the risk of recurrence [hazard ratio (HR) = 1.40, confidence interval (CI) = (1.12–1.74); *P* = 0.003] and with a 58% increase in the risk of death [HR = 1.58, CI = (1.21–2.06); *P* < 0.001]. Upon doubling of the MACC1 level, we observed a 39% increase in the risk of recurrence [HR = 1.39, CI = (1.09–1.77); *P* = 0.009] and a 53% increase in the risk of death [HR = 1.53, CI = (1.10–2.13); *P* = 0.012; Fig. S2].

An increase of 1 relative unit for S100A4 was associated with a 0.2% increase in the risk of recurrence [HR = 1.002, CI = (1.0006–1.003); *P* = 0.001] and with a 0.1% increase in the risk of death [HR = 1.001, CI = (1.0005–1.002); *P* = 0.002]. Upon doubling of the S100A4 level, we observed a 37% increase in the risk of recurrence [HR = 1.37, CI = (1.08–1.74); *P* = 0.01] and a 43% increase in the risk of death [HR = 1.43, CI = (1.03–1.99), *P* = 0.035; Fig. S2].

**Figure 4:**
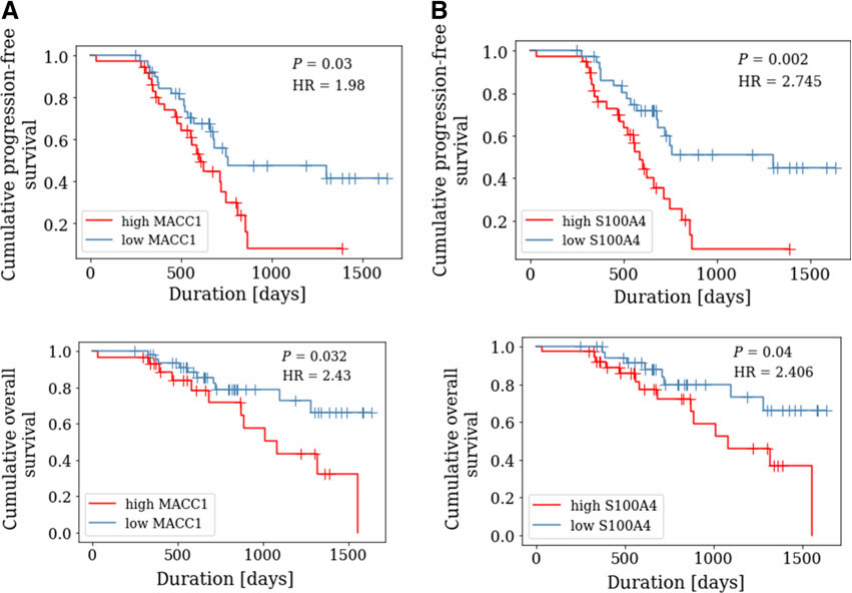
Prognostic relevance of circulating MACC1 and S100A4 transcripts at primary diagnosis. Kaplan–Meier analysis comparing progression‐free survival (PFS) and overall survival (OS) of ovarian cancer patients with high levels of circulating MACC1 (A) or S100A4 (B) transcripts vs. low levels of circulating MACC1 or S100A4 transcripts. High MACC1 levels were defined as a MACC1 value > 0.776%calibrator (for PFS analysis) and as > 0.946% calibrator (for OS analysis). High S100A4 levels were defined as a S100A4 value above the median (for PFS and OS analysis). *P*‐values, according to log‐rank test and hazard ratio (HR), are indicated.

Additionally, Kaplan–Meier analysis and the log‐rank test were performed. Patients with high levels of circulating MACC1 transcripts had a significantly decreased PFS [*P* = 0.03, HR = 1.98, CI = (1.06–3.72)] and a decreased OS [*P* = 0.032, HR = 2.43, CI = (1.05–5.64)] compared to patients with low MACC1 levels (Fig. 4A). Accordingly, patients with high levels of circulating S100A4 transcripts had a significantly decreased PFS [*P* = 0.002, HR = 2.74 CI = (1.39–5.02)] and a significantly decreased OS [*P* = 0.039, HR = 2.41, CI = (1.02–5.84)] compared to patients with low S100A4 levels (Fig. 4B).

We additionally performed multivariate Cox regression analysis with PFS or OS as selected outcome variables, including MACC1 and S100A4 levels and established risk factors of ovarian cancer, that is, age, presence of malignant ascites, tumor stage, histology and general state of health, according to the risk classification of the American Society of Anesthesiologists (ASA). We could not confirm that MACC1 and S100A4 transcript levels are independent predictors of decreased PFS and OS (Fig. S3).

Subsequently, we analyzed prognostic relevance of MACC1 and S100A4 levels in the longitudinal follow‐up samples. Considering the level of MACC1 and S100A4 independently at each reading point of blood sampling (thus disregarding the individual time‐dependent course), their levels among all follow‐up samples did not provide prognostic information (Fig. S2). In a more detailed analysis, we performed univariate Cox regression analysis, now incorporating MACC1 or S100A4 values as time‐dependent variables across all reading points of blood drawing. The time‐dependent course of MACC1 and S100A4 transcripts levels did not predict risk of recurrence or risk of death.

**Figure 5:**
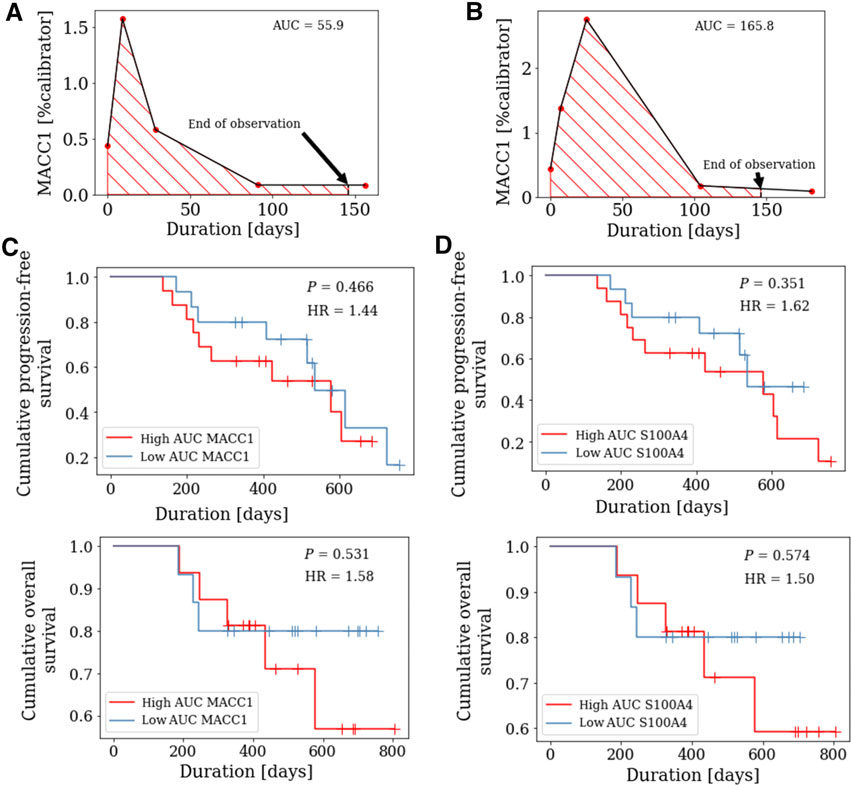
Prognostic relevance of circulating MACC1 and S100A4 transcripts in the course of adjuvant chemotherapy. Assuming a linear change of MACC1 or S100A4 values, ‘dynamic curves’ were plotted for each patient, reflecting the individual course of MACC1 or S100A4 for all patients with a complete set of blood samples (*n* = 31) in the course of surgery and adjuvant chemotherapy. From each plot, the area under the curve (AUC) was calculated for an observation time of 146 days (which was an available time period for all included patients) and was applied for Kaplan–Meier analysis. The Figure shows two representative ‘dynamic curves’ for circulating MACC1 transcripts with a low AUC (defined as an AUC value below the median level, (A) vs. high AUC (defined as an AUC value equal or above the median level, (B). Kaplan–Meier curves were drawn, comparing (C) progression‐free survival (PFS) and (D) overall survival (OS) of ovarian cancer patients with high AUC of MACC1 or S100A4, respectively, vs. patients with low AUC of MACC1 or S100A4, respectively. *P*‐values, according to log‐rank test, are indicated.

Finally, assuming a linear change of MACC1 or S100A4 values between the investigated follow‐up reading points, we plotted for each patient a dynamic curve by interconnecting the values for MACC1 and S100A4, thus reflecting the individual course of MACC1 or S100A4 for each patient during surgery and chemotherapy (Fig. 5A + B). From each curve, the AUC was calculated and then applied for Kaplan–Meier analysis. Therefore, all patients were stratified into a high‐AUC group (defined as an AUC value above the median level) and into a low‐AUC group (defined as an AUC value below the median level). There was no statistically significant difference between MACC1 AUC high vs. low patients (Fig. 5C) or between S100A4 high vs. low patients (Fig. 5D).

We conclude that high levels of circulating MACC1 or S100A4 transcripts at primary diagnosis (but not among follow‐up samples during surgery and adjuvant chemotherapy) are unfavorable prognostic factors for ovarian cancer patients and indicate poor prognosis.

## 4 Discussion

In the present liquid biopsy approach, we systematically analyzed clinical relevance of circulating MACC1 and S100A4 transcripts in ovarian cancer patients. We report that high serum levels of both transcripts at primary diagnosis (but not in the course of chemotherapy) are associated with advanced disease, predict suboptimal primary debulking, and indicate poor survival.

The mechanisms, how nucleic acids are released into the bloodstream, are still unclear. However, robust data suggest that fragmented tumor DNA or RNA found in plasma is derived mostly from apoptotic cancer cells or by active secretion mechanism involving microvesicles, such as exosomes (Schwarzenbach, 2015; Schwarzenbach *et al*., 2011). We observed that serum levels of both, MACC1 and S100A4, are significantly upregulated at primary diagnosis of ovarian cancer, compared to healthy controls. This is in accordance with our previous studies on CRC, in which MACC1 was shown to be significantly upregulated either in malignant CRC tissue vs. healthy colonic mucosa or in plasma from CRC patients vs. plasma from healthy controls (Ashktorab *et al*., 2016; Stein *et al*., 2009a). The same holds true when comparing MACC1 levels in plasma from gastric cancer patients compared to healthy controls (Ashktorab *et al*., 2016; Burock *et al*., 2015; Stein *et al*., 2012). Moreover, we have also observed upregulation of S100A4 transcripts in plasma from patients with CRC or gastric cancer (Stein *et al*., 2011, 2012). Our data now extend our knowledge on MACC1 and S100A4 to ovarian cancer.

In contrast to MACC1, S100A4 levels in serum of ovarian cancer patients showed only a small overlap to healthy controls, resulting in a good discrimination between ‘ovarian cancer’ and ‘healthy’ (AUC = 0.81). Future studies will have to address, whether the circulating S100A4 transcript level could be used as a blood‐based screening marker for ovarian cancer. Therefore, independent patient cohorts with low‐stage ovarian cancer (FIGO I‐II) will be necessary.

The levels of circulating MACC1 or S100A4 transcripts showed a characteristic and highly concordant dynamic in the course of primary surgery and chemotherapy. Both transcript levels strongly increased at the measure point one week after primary debulking surgery. However, this increase did neither correlate with debulking efficiency nor with residual tumor burden or prognosis, suggesting that this phenomenon could be caused by an unspecific and transient release of MACC1 and S100A4 mRNA into the blood, possibly due to tissue damage during surgery.

Applying different PCR‐based methods for MACC1 and S100A4 detection, we report that circulating MACC1 and S100A4 transcript levels strongly correlate at all investigated reading points. Although MACC1 and S100A4 are involved in different biological pathways, that is, MACC1 as a key regulator of HGF‐MET signaling (Stein *et al*., 2009a) and S100A4 as a transcriptional target of β‐catenin/T‐cell factor signaling (Stein *et al*., 2006), our data suggest that MACC1 and S100A4 might be co‐regulated in cancer. Further, since MACC1 is reported to regulate Wnt/β‐catenin signaling (Zhen *et al*., 2014), also a direct regulation of S100A4 as a Wnt/β‐catenin signaling target cannot be excluded. Therefore, the highly concordant dynamics of MACC1 and S100A4 transcripts in serum of ovarian cancer patients could be explained by a proportional release upon apoptosis or by a possibly co‐regulated active secretion.

Since we found that levels of MACC1 and S100A4 transcripts at primary diagnosis correlated with FIGO stage, we suggest that a significant proportion of MACC1 and S100A4 transcripts is derived from ovarian cancer cells and reflects the patient's individual tumor load. This is further supported by our finding that high serum levels were associated with suboptimal debulking surgery. We hypothesize that high serum levels of these transcripts are associated with advanced tumors with aggressive and complex growth patterns, which are more difficult to be resected.

The strength of our study is that we analyzed serum MACC1 and S100A4 transcripts not only at primary diagnosis but also in an extended serum set of four longitudinal follow‐up samples per patient in the course of surgery and chemotherapy. Taking advantage of these extended serum sets, we can now draw the substantiated conclusion that high MACC1 and S100A4 serum levels at primary diagnosis have prognostic relevance and indicate poor survival. Since serum levels of MACC1 and S100A4 were independent from CA125 levels, they could be useful as auxiliary liquid biopsy markers for stratifying prognosis. Such an unfavorable prognostic impact of high MACC1 and S100A4 expression at primary diagnosis has already been reported by our group and by independent investigators for a variety of other solid cancer entities, including CRC, gastric cancer, pancreatic cancer or glioblastoma (Burock *et al*., 2015; Hagemann *et al*., 2013; Huang *et al*., 2016; Kikuchi *et al*., 2006; Stein *et al*., 2009a); this either among primary‐tumor or blood‐based approaches. Nevertheless, despite a very recent preliminary study, investigating clinical relevance of S100A4 protein in serum (Lv *et al*., 2018), this is the first study on MACC1 and S100A4 serum transcripts in ovarian cancer. As a limitation of our study, a complete series of longitudinal follow‐up samples was only available from a part of our patients (*n* = 31) and not from the entire cohort.

We encourage further investigation, considering MACC1 and S100A4 as a potential therapeutic target for ovarian cancer. In this context, we have already designed pharmacological strategies for inhibiting MACC1 by the small‐molecule transcriptional inhibitors lovastatin or rottlerin for CRC (Juneja *et al*., 2017). Moreover, we showed that the antihelminthic drug niclosamide inhibits S100A4 transcription and we are currently performing a clinical phase II trial on stage IV CRC, in which niclosamide is applied in terms of a drug repositioning approach (EudraCT 2014‐005151‐20, NCT02519582). Future studies will have to address whether this concept could be transferred to ovarian cancer.

## 5 Conclusions

We report that high MACC1 and S100A4 transcript levels at primary diagnosis of ovarian cancer are associated with advanced disease and predict poor prognosis. Our data harmonize with the previously shown oncogenic and metastasis‐initiating impact of MACC1 and S100A4 and extend our knowledge of these genes to innovative liquid biopsy approaches for ovarian cancer. Furthermore, we encourage future translational investigations with MACC1 and S100A4 as therapeutic targets for ovarian cancer.

## Conflict of interest

The authors declare no conflict of interest.

## Author contributions

TL, JDK, MK, PW, and US made substantial contributions to the conception and design of the study. DK, PH, YV, MK, KF, MG, US, TL, and JDK to the experimental work, to the acquisition of data, and to the analysis/interpretation of the results. YDV performed statistical analysis under supervision of MK. JDK, US, PW, TL, and DK were involved in drafting the manuscript or revising it. All authors read and approved the manuscript in its final version.

## Supporting information


**Fig. S1.** Discrimination of circulating MACC1 and S100A4 transcripts between ovarian cancer patients and healthy controls. ROC curve analysis was performed to assess, whether circulating MACC1 or S100A4 serum levels at primary diagnosis enable discrimination between ovarian cancer patients and healthy controls. The corresponding area under the curve (AUC) values are indicated.
**Fig. S2.** Prognostic relevance of circulating MACC1 and S100A4 transcripts at primary diagnosis and in the course of treatment. An univariate Cox regression analysis was performed. The figure shows the natural logarithm of the hazard ratios (upon doubling of the MACC1 or S100A4 levels) and the corresponding confidence intervals.
**Fig. S3.** Prognostic relevance of circulating MACC1 and S100A4 transcripts at primary diagnosis in relation to established risk factors of ovarian cancer. A multivariate Cox regression analysis was performed, adjusted for established ovarian cancer risk factors. The figure shows the natural logarithm of the hazard ratios and the corresponding confidence intervals.
