# Supplementary material for: Clinical relevance of circulating MACC1 and S100A4 transcripts for ovarian cancer
Source: Mol Oncol. 2019 Apr 15;13(5):1268–79. doi: 10.1002/1878-0261.12484 (PMC6487687; doi:10.1002/1878-0261.12484)
Supplement: Supplementary file 1 — Fig. S1. Discrimination of circulating MACC1 and S100A4 transcripts between ovarian cancer patients and healthy controls. ROC curve analysis was performed to assess, whether circulating MACC1 or S100A4 serum levels at primary diagnosis enable discrimination between ovarian cancer patients and healthy controls. The corresponding area under the curve (AUC) values are indicated. Fig. S2. Prognostic relevance of circulating MACC1 and S100A4 transcripts at primary diagnosis and in the course of treatment. An univariate Cox regression analysis was performed. The figure shows the natural logarithm of the hazard ratios (upon doubling of the MACC1 or S100A4 levels) and the corresponding confidence intervals. Fig. S3. Prognostic relevance of circulating MACC1 and S100A4 transcripts at primary diagnosis in relation to established risk factors of ovarian cancer. A multivariate Cox regression analysis was performed, adjusted for established ovarian cancer risk factors. The figure shows the natural logarithm of the hazard ratios and the corresponding confidence intervals. [file MOL2-13-1268-s001.pdf]

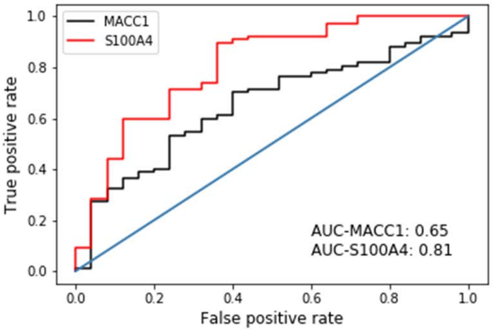

MACC1
S100A4
True positive rate
False positive rate
AUC-MACC1: 0.65
AUC-S100A4: 0.81
0.0
0.2
0.4
0.6
0.8
1.0

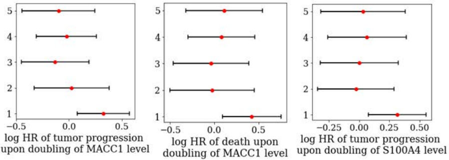

1 = patients at primary diagnosis
2 = patients postoperative
3 = patients before CTx
4 = patients after third cycle CTx
5 = patients after CTx

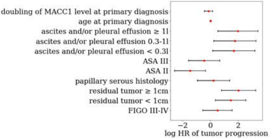
doubling of MACC1 level at primary diagnosis
age at primary diagnosis
ascites and/or pleural effusion ≥ 1l
ascites and/or pleural effusion 0.3-1l
ascites and/or pleural effusion < 0.3l
ASA III
ASA II
papillary serous histology
residual tumor ≥ 1cm
residual tumor < 1cm
FIGO III-IV
−2
0
2
log HR of tumor progression

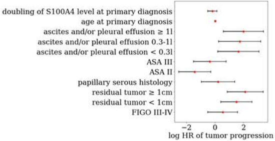
doubling of S100A4 level at primary diagnosis
age at primary diagnosis
ascites and/or pleural effusion ≥ 1l
ascites and/or pleural effusion 0.3-1l
ascites and/or pleural effusion < 0.3l
ASA III
ASA II
papillary serous histology
residual tumor ≥ 1cm
residual tumor < 1cm
FIGO III-IV
−2
0
2
log HR of tumor progression

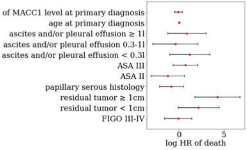
doubling of MACC1 level at primary diagnosis
age at primary diagnosis
ascites and/or pleural effusion ≥ 1l
ascites and/or pleural effusion 0.3-1l
ascites and/or pleural effusion < 0.3l
ASA III
ASA II
papillary serous histology
residual tumor ≥ 1cm
residual tumor < 1cm
FIGO III-IV
0
5
log HR of death

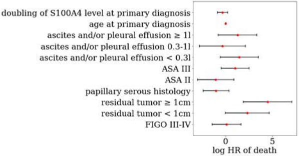
doubling of S100A4 level at primary diagnosis
age at primary diagnosis
ascites and/or pleural effusion ≥ 1l
ascites and/or pleural effusion 0.3-1l
ascites and/or pleural effusion < 0.3l
ASA III
ASA II
papillary serous histology
residual tumor ≥ 1cm
residual tumor < 1cm
FIGO III-IV
0
5
log HR of death
